# Utilization of Carbon Fiber Spinal Instrumentation in Primary Spinal Tumors: Outcome Analysis from the AO Spine Primary Tumor Research and Outcomes Network

**DOI:** 10.1177/21925682261417978

**Published:** 2026-09-13

**Authors:** Gisberto Evangelisti, Marie-Laure Vial, Feng Wei, Alessandro Gasbarrini, Laurence D. Rhines, Ziya L. Gokaslan, Jeremy Reynolds, Alessandro Luzzati, Alexander C. Disch, Praveen V. Mummaneni, Daniel G. Tobert, Michelle J. Clarke, Aron Lazary, Chetan Bettegowda, Riccardo Cecchinato, Stefano Boriani, Ori Barzilai, Cordula Netzer, Arjun Sahgal, Jorrit-Jan Verlaan, Charles G. Fisher, Ilya Laufer, Nicolas Dea, on behalf of AO Spine Knowledge Forum Tumor

**Affiliations:** 1 Department of Spine Surgery, 18509 IRCCS Istituto Ortopedico Rizzoli, Bologna, Italy; 2 AO Innovation Translation Center, AO Foundation, Davos Platz, Switzerland; 3 Department of Orthopaedic, 66482 Peking University Third Hospital, Beijing, China; 4 Department of Biomedical and Neuromotor Sciences, 9296 University of Bologna, Bologna, Italy; 5 Department of Neurosurgery, 461926 The University of Texas MD Anderson Cancer Center, Houston, TX, USA; 6 Department of Neurosurgery, 12321 The Warren Alpert Medical School of Brown University, Providence, RI, USA; 7 6397 Oxford University Hospitals NHS Trust, Oxford, UK; 8 Department of Orthopaedics, 46767 IRCCS Istituto Ortopedico Galeazzi, Milan, Italy; 9 University Comprehensive Spine Center, 39063 University Hospital Carl Gustav Carus, Dresden, Germany; 10 Department of Neurosurgery, 8785 University of California, San Francisco, CA, USA; 11 Department of Othopedic Surgery, 2348 Massachusetts General Hospital, Boston, MA, USA; 12 Department of Neurosurgery, 6915 Mayo Clinic, Rochester, MN, USA; 13 National Center for Spinal Disorders, Buda Health Center, Budapest, Hungary; 14 Department of Neurosurgery, 536756 Johns Hopkins University School of Medicine, Baltimore, MD, USA; 15 Department of Biomedical Sciences for Health, University of Milan, Milan, Italy; 16 46767 IRCCS Galeazzi-Sant’Ambrogio Hospital, Milan, Italy; 17 Post Graduate Program of Orthopedics, 9296 University of Bologna, Bologna, Italy; 18 Department of Neurosurgery, 5803 Memorial Sloan Kettering Cancer Center, New York, NY, USA; 19 Department of Spine Surgery, 30262 University Hospital of Basel, Basel, Switzerland; 20 Departments of Radiation Oncology and Surgery, University of Toronto, Toronto, ON, Canada; 21 Department of Orthopaedics, 8124 University Medical Center Utrecht, Utrecht, The Netherlands; 22 Combined Neurosurgical and Orthopaedic Spine Program, University of British Columbia, Vancouver, BC, Canada; 23 Department of Neurosurgery, 12297 NYU Langone Medical Center, New York, NY, USA

**Keywords:** complications, primary spinal tumor, carbon fiber implants

## Abstract

**Background:**

The use of carbon fiber-reinforced polyetheretherketone (CFR-PEEK) instrumentation in spinal surgery has emerged as an alternative to traditional titanium-based implants in recent years. This study aims to evaluate the use and performance of carbon fiber instrumentation in patients with primary spinal tumors focusing on adverse event (AE) risk, overall survival and oncologic outcomes.

**Methods:**

Data were collected from the AO Spine Primary Tumor Research and Outcomes Network (PTRON), a multicenter international registry. The primary endpoint was the incidence of AEs, while secondary endpoints included the risk of developing at least one AE, overall survival, local tumor control, and disease progression.

**Results:**

A total of 359 patients enrolled in the PTRON registry met the inclusion criteria and were included in the study. Among them, 84 receiving carbon fiber implants (23%), 243 titanium implants (68%), and 32 a combination of both (9%). Median follow-up times were 1.9 years (IQR: 0.4-3.0), 1.3 years (IQR: 0.5-2.4) and 1.1 years (IQR: 0.5-1.7), in the carbon fiber, titanium and combination groups, respectively. The estimated risk of having at least one postoperative AE, including construct failures with and without loss of correction, wound infection (deep or superficial), non-union and wound dehiscence, was statistically comparable between patients receiving carbon fiber, titanium or a combination of both carbon fiber and titanium implants (*P* > 0.05). Kaplan-Meier survival analyses and multivariable Cox proportional hazards models showed no significant differences in overall survival, recurrence-free survival, or progression-free survival between the implant groups.

**Conclusion:**

Carbon fiber spinal implants demonstrate a comparable safety and oncologic profile to traditional titanium implants while allowing for improved tumor surveillance and easier radiation delivery.

## Introduction

Traditionally, titanium-based instrumentation has been the standard for spinal fixation following tumor resection.^1^ However, the use of metallic implants can pose challenges in post-operative imaging surveillance and radiation therapy planning, which are crucial in the management of spinal tumors.^2,3^

In recent years, carbon fiber reinforced polyetheretherketone (CFR-PEEK) instrumentation has emerged as an alternative to traditional metallic implants in spinal surgery. Carbon fiber components offer several potential advantages in the context of spinal oncology.^4^ Their radiolucent properties allow for improved visualization in post-operative imaging, facilitating better assessment of tumor recurrence or progression.^1,4,5^ Moreover, carbon fiber implants produce fewer artifacts in computed tomography (CT) and magnetic resonance imaging (MRI) scans, enabling more accurate radiotherapy planning and delivery.^4,6–8^ In addition, CFR-PEEK implants have a modulus of elasticity closer to that of cortical bone compared to traditional metallic implants, potentially reducing stress shielding and promoting a more favorable environment for bone fusion.^8^

Despite these potential benefits, the use of carbon fiber instrumentation in primary spinal tumors remains a relatively new and evolving field. Questions persist regarding its long-term durability, biomechanical performance under various loading conditions, and its impact on overall patient outcomes.^8^

The complex biomechanical environment created by tumor resection, coupled with the potential need for adjuvant therapies, necessitates a thorough evaluation of the efficacy and safety profile of carbon fiber instrumentation in this specific patient population.

To address these knowledge gaps, this study aims to comprehensively evaluate the use and performance of carbon fiber instrumentation in patients with primary spinal tumors, using data from the AO Spine Primary Tumor Research and Outcomes Network (PTRON), a multicenter international registry with prospectively collected data from patients with primary spine tumors.

The primary objective of this study was to compare the incidence of adverse events (AEs) among patients receiving carbon fiber implants, titanium implants, or a combination of both.

Secondary objectives included comparison of the risk of AEs among the three implant groups and evaluation of the impact of carbon fiber implants or a combination of carbon fiber and titanium implants compared to titanium implants alone, on overall survival and disease outcomes – including local recurrence and progression (metastases)– using overall survival and event-free survival analyses with a combined endpoint approach in patients with primary spinal tumors.

## Material and Methods

### Study Design and Inclusion Criteria

The Primary Tumor Research and Outcomes Network (PTRON) is a multicenter registry that prospectively collects data from patients with primary spine tumors (ClinicalTrials.gov ID: NCT02790983). Fifteen international centers with expertise in spine oncology are currently open in this registry and have received approval from their local ethics board.

Inclusion criteria were: (1) patients diagnosed with a primary tumor of the spine and enrolled in the PTRON registry; and (2) use of carbon fiber and/or titanium instrumentation (including cages, anterior or posterior plates, screws, or rods) for spinal fixation. Exclusion criteria were: (1) patients diagnosed with metastatic spinal tumors or primary spinal cord tumors; and (2) patients with missing information regarding the implant material. Written informed consent was obtained from all patients included in the study. Available data from February 27, 2017, to March 28, 2025, were analyzed. While details on cage material have been collected since the inception of PTRON, information on anterior and posterior fixation types has only been systematically recorded since March 2023.

### Data Collection

Patient demographics including sex, age, body mass index (BMI), ethnicity, and Charlson Comorbidity index (CCI) were collected. Additionally, information on tumor type, tumor resection scenario, and adjuvant radiation therapy were also recorded.

### Data Analysis

Differences between patients with a carbon fiber, a titanium or a combination of carbon fiber and titanium implants were investigated.

The primary endpoint was incidence of AEs, defined as time to the first AE, analyzed by implant type.

Secondary endpoints included the risk of developing at least one AE, overall survival, recurrence-free survival and progression-free survival, all analyzed by implant type.

Simple summary statistics were used for summarizing patient demographics, tumor types, adjuvant radiation therapy administration and tumor resection scenarios by implant type. Categorical variables were summarized using the frequency and percentage for each category. Continuous variables were summarized using n, mean, standard deviation (sd), median, lower and upper values of the inter-quartile range, and minimum and maximum values. Chi2-test was used to compare categorical variables between the implant groups. If the expected cell count was less than five in one or more of the cells, the Fisher’s exact test was used for the analyses.

Time to the first AE was investigated using time-to-event analyses. We defined remaining free from the first AE as the time from the first implant (for patients receiving either a combination of carbon fiber and titanium implants or multiple implants of the same type) until the occurrence of the first AE, death, dropout or the last follow-up visit whichever occurred first. Kaplan-Meier survival curves were plotted to estimate the probability of remaining free from a first AE. The Kaplan Meier curves were compared with the use of a log-rank test. Hazard ratios (HR) for the risk of an AE were estimated using univariate and multivariable Cox proportional hazards regression models. The multivariable model was adjusted for sex, age, tumor type and adjuvant therapy as radiation therapy treatment administration. Predictor selection was based on the Akaike Information Criterion (AIC) and Bayesian Information Criterion (BIC) and on internal discussions.

Summary tables on AEs were produced by category at patient level. Events of the same type were combined for each patient, thus counting each patient with at least one AE only once. When calculating event rates, the denominators were the total population size, irrespective of dropouts during follow-up. This approach assumed that any AEs was reported, even after a patient was lost to follow-up. Event rates were calculated with 95% CI using the Clopper-Pearson method.

For all the analyses, AEs were considered if they occurred at the date or after the date of a carbon fiber or titanium implant.

Overall survival, recurrence-free survival and progression-free survival (metastases) were also investigated using time-to-event analyses. We defined overall survival as time from the first implant (for patients receiving either a combination of carbon fiber and titanium implants or multiple implants of the same type) until death, dropout or the last follow-up visit whichever occurred first. We defined recurrence-free survival and progression-free survival as the time from the first implant (for patients receiving either a combination of carbon fiber and titanium implants or multiple implants of the same type) until the first occurrence of local recurrence or progression (metastases) respectively or until death, dropout or the last follow-up visit whichever occurred first.

For the recurrence-free survival and progression-free survival analyses, we used a combined endpoint approach treating both local recurrence and death, and progression (metastases) and death, as events. Within sensitivity analyses, we also applied the event-free approach without a combined endpoint to evaluate the robustness of our findings. The very small sample size for patients with a combination of carbon fiber and titanium implants did not allow for analyses using a competing risk approach.

Kaplan-Meier survival curves were used to estimate the probabilities of overall survival and remaining free from recurrence or progression (metastases). The Kaplan-Meier curves were compared with the use of a log-rank test. Finally, HR for the risk of death, local recurrence and for progression (metastases) were estimated using univariate and multivariable Cox proportional hazards regression models. The models were adjusted for the same predictors as the multivariable model used in the AE incidence analysis.

All analyses were performed using SAS Software version 9.4 (SAS, North Carolina, USA).

## Results

### Patient Demographics

A total of 359 patients enrolled in the PTRON registry met the inclusion criteria and were included in the study. Among them, 84 receiving carbon fiber implants (23%), 243 titanium implants (68%), and 32 a combination of both (9%). The sex distribution showed that 154 patients were female (42.9%) and 205 patients were male (57.1%). Male patients constituted the majority across all groups, with the highest proportion in the combination group (75.0%), followed by the carbon fiber group (60.7%) and the titanium group (53.5%).

The overall average age was 47.5 years (±18.6). Patients in the combination group (54.8 ± 18.1 years) were, on average, older than those in the carbon fiber (51.2 ± 18.8 years) or the titanium (45.2 ± 18.8 years) groups. Over two-thirds of the patients were Caucasians (70.5%), and a small proportion were pediatric patients (3.6%). The overall average BMI was 26.4 (±5.2) and was comparable between the groups, with a slightly higher value observed in the combination group (28.5) (±5.3) compared to the carbon fiber group (26.3 ± 4.8) and the titanium group (26.2 ± 5.3). The Charlston Comorbidity Index was 2.0 (±1.1), 2.1 (±1.6) and 2.3 (±0.7) for the carbon fiber, titanium and combination groups, respectively (Table 1).

**Table 1.** Summary of Patient Demographics by Implant Type (Eligible and Enrolled Patients)

Variable	Implant			
	Carbon fiber implant N = 84	Titanium implant N = 243	Combination of carbon fiber and titanium implants N = 32	Total N = 359
Sex, n (%)	84	243	32	359
Female	33 (39.3)	113 (46.5)	8 (25.0)	154 (42.9)
Male	51 (60.7)	130 (53.5)	24 (75.0)	205 (57.1)
Age (years)				
n	84	243	32	359
Mean (sd)	51.2 (18.8)	45.2 (18.1)	54.8 (18.9)	47.5 (18.6)
Median (Q1; Q3)	55.0 (35.0; 65.5)	44.0 (30.0; 62.0)	56.5 (43.0; 69.0)	49.0 (31.0; 64.0)
Min; Max	15.0; 84.0	4.0; 82.0	7.0; 81.0	4.0; 84.0
Pediatric and adult patients, n (%)	84	243	32	359
Pediatric (<18 yrs)	1 (1.2)	11 (4.5)	1 (3.1)	13 (3.6)
Adult (>= 18 yrs)	83 (98.8)	232 (95.5)	31 (96.9)	346 (96.4)
BMI				
n	82	239	32	353
Mean (sd)	26.3 (4.8)	26.2 (5.3)	28.5 (5.3)	26.4 (5.2)
Median (Q1; Q3)	25.9 (22.5; 29.4)	25.7 (22.6; 29.2)	28.0 (25.2; 31.6)	26.0 (22.7; 29.5)
Min; Max	15.6; 39.7	14.7; 49.2	16.3; 40.5	14.7; 49.2
Ethnicity, n (%)	84	243	32	359
Caucasian	72 (85.7)	151 (62.1)	30 (93.8)	253 (70.5)
African/Black	2 (2.4)	4 (1.6)	0 (0.0)	6 (1.7)
Asian/Pacific Islander	3 (3.6)	76 (31.3)	0 (0.0)	79 (22.0)
Aboriginal	0 (0.0)	0 (0.0)	0 (0.0)	0 (0.0)
Hispanic	3 (3.6)	5 (2.1)	2 (6.3)	10 (2.8)
Caribbean	0 (0.0)	0 (0.0)	0 (0.0)	0 (0.0)
East Indian	1 (1.2)	0 (0.0)	0 (0.0)	1 (0.3)
Other/Mixed	2 (2.4)	4 (1.6)	0 (0.0)	6 (1.7)
Unknown	1 (1.2)	3 (1.2)	0 (0.0)	4 (1.1)
Charlson comorbidity index				
n	84	243	32	359
Mean (sd)	2.0 (1.1)	2.1 (1.6)	2.3 (0.7)	2.1 (1.5)
Median (Q1; Q3)	2.0 (2.0; 2.0)	2.0 (2.0; 2.0)	2.0 (2.0; 3.0)	2.0 (2.0; 2.0)
Min; Max	0.0; 7.0	0.0; 12.0	0.0; 4.0	0.0; 12.0

### Overview of Primary Tumor Types and Treatment Strategies

Median follow-up times were 1.9 years (IQR: 0.4-3.0), 1.3 years (IQR: 0.5-2.4) and 1.1 years (IQR: 0.5-1.7), in the carbon fiber, titanium and combination groups, respectively. The maximum follow-up time was 7.6 years, which involved a patient with a titanium implant.

The distribution of primary tumor groups differed significantly among the carbon fiber, titanium, and combination implant groups (*P* < 0.001). Chordoma was the most common diagnosis among patients who received a carbon fiber implant (45.2%), whereas high-grade tumors were the most frequent among those who received a titanium implant (28.1%). Among patients with a combination of carbon fiber and titanium implants, chordoma and high-grade tumors were equally the most common diagnoses (43.8% each) (Table 2).

**Table 2.** Tumor Type Groups, Radiation Therapy Treatment Administration and Surgical Resection Scenarios by Implant Type

Variable	Implant				*P* value
	Carbon fiber implant N = 84	Titanium implant N = 243	Combination of carbon fiber and titanium implant N = 32	Total N = 359	
Tumor type groups, n (%)	84	242	32	358	<.001^a^
Benign (latent/active)	4 (4.8)	32 (13.2)	0 (0.0)	36 (10.1)	
Benign (aggressive)	12 (14.3)	57 (23.6)	2 (6.3)	71 (19.8)	
Malignant	23 (27.4)	68 (28.1)	14 (43.8)	105 (29.3)	
Chordoma	38 (45.2)	43 (17.8)	14 (43.8)	95 (26.5)	
Other tumor type groups	7 (8.3)	42 (17.4)	2 (6.3)	51 (14.2)	
Treatment administration: Adjuvant therapy, n (%)	84	238	32	354	0.004^a^
No	65 (77.4)	199 (83.6)	23 (71.9)	287 (81.1)	
Yes	19 (22.6)	39 (16.4)	9 (28.1)	67 (18.9)	
Surgical resection scenarios, n (%)	41	148	25	214	<.001^a^
Planned transgression	0 (0.0)	0 (0.0)	0 (0.0)	0 (0.0)	
True en bloc	26 (63.4)	77 (52.0)	18 (72.0)	121 (56.5)	
Deliberate Intralesional	11 (26.8)	49 (33.1)	4 (16.0)	64 (29.9)	
En bloc resection after Intralesional surgery	3 (7.3)	4 (2.7)	3 (12.0)	10 (4.7)	
Failed en bloc	1 (2.4)	18 (12.2)	0 (0.0)	19 (8.9)	

^a^Fisher’s exact test.

The low-grade group comprises Benign notochordal cell tumor (BNCT), Osteochondroma (Exostosis), Enchondroma, Osteoid osteoma, Langerhans cell histiocytosis (LCH) (eosinophilic), Fibrous dysplasia, Unicameral bone cyst, Chondroblastoma, Schwannoma (Benign peripheral nerve sheath tumor) and Neurofibroma.

The locally invasive group comprises Giant cell tumor, Osteoblastoma, Hemangioma, Aneurysmal bone cyst and Epithelioid Hemangioma.

The malignant group comprises Chondrosarcoma, Osteosarcoma, Ewing’s sarcoma, Undifferentiated high grade pleomorphic sarcoma, Malignant peripheral nerve sheath tumor, Hemangiopericytoma/solitary fibrous tumor, Angiosarcoma and Epithelioid Hemangioendothelioma.

Surgical resection data were unavailable for 93 patients and therefore were not included in the table.

Although the overall distribution of resection types also varied significantly across implant groups (*P* < 0.001), a true en bloc resection (en bloc resection with negative margin) remained the most common surgical approach in all groups, observed in 63.4% of patients in the carbon fiber group, 52.0% in the titanium group, and 72.0% in the combination group. A total of 19 failed en bloc resections (en bloc resection with positive margin) were recorded, the majority of which occurred in the titanium implant group (n = 18, 12.2%) (Table 2).

The proportion of patients who received adjuvant radiation therapy differed significantly among the carbon fiber, titanium, and combination groups (*P* = 0.004), with rates of 22.6%, 16.4%, and 28.1%, respectively (Table 2).

### Incidence and Risk Analysis of Adverse Events

**Figure 1.**
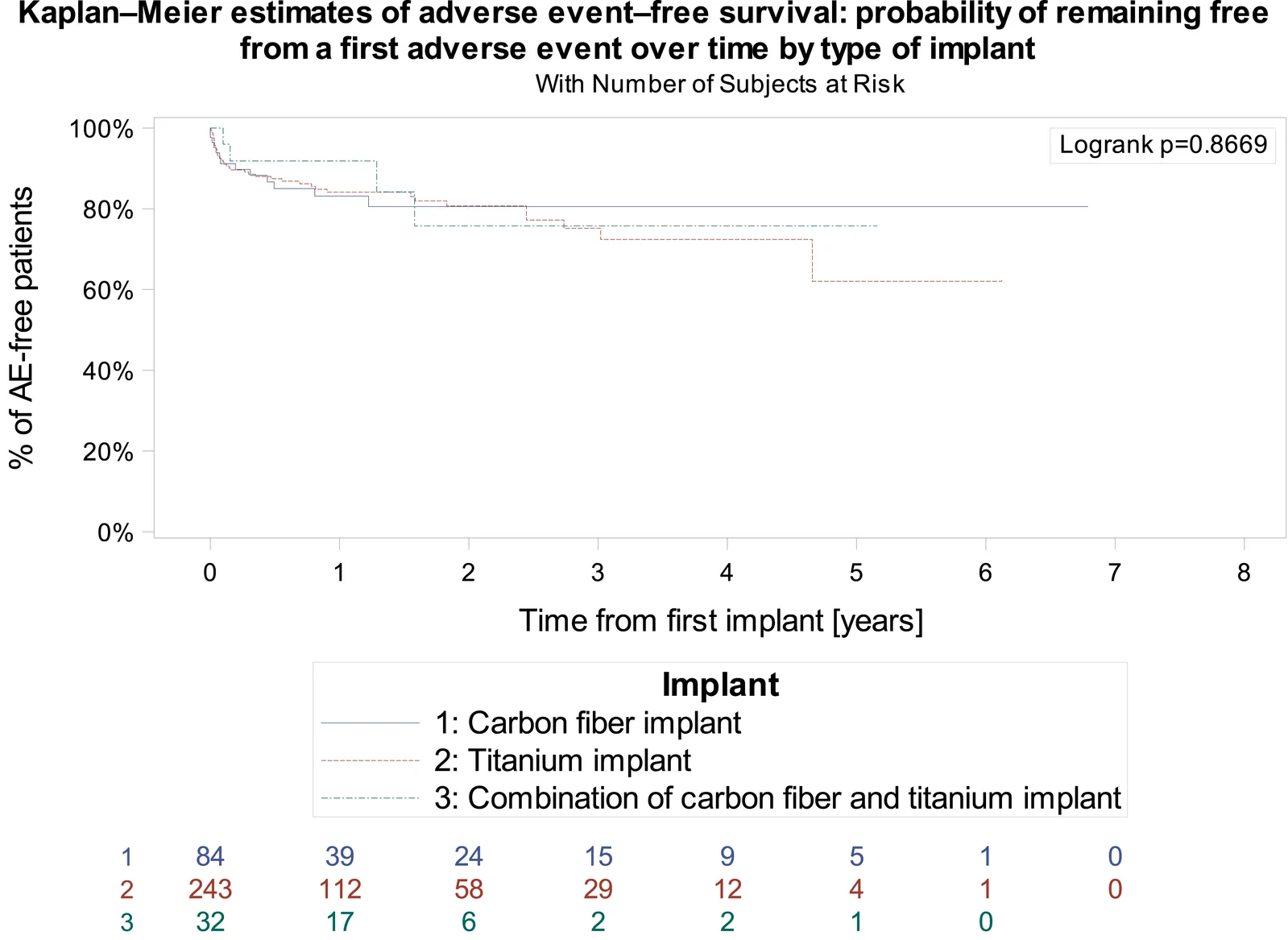
Kaplan–Meier survival curve estimating the probability of remaining free from a first adverse event over time by implant type

Kaplan-Meier curve analysis demonstrated no significant difference in the probability of remaining free from AEs over time across all implant groups (log-rank *P* = 0.867) (Figure 1).

Univariate and multivariable Cox proportional hazards analyses also showed no significant difference in the risk of experiencing a first AE for patients with a carbon fiber or a combination of carbon fiber and titanium implants compared to those with a titanium implant only. Compared to titanium (reference), HR estimated from the multivariable Cox proportional hazards regression models were 0.53 (95% CI: 0.26-1.07, *P* = 0.08) for carbon fiber and 0.57 (95% CI: 0.20-1.64, *P* = 0.30) for the implant combination group (Table 3).

**Table 3.** Time-To-Event Analysis of Adverse Event-Free Survival by Implant Group

Outcome	Implant			
	Carbon fiber implant	Titanium implant	Combination of carbon fiber and titanium implants	Summary/*P* value
Total number of patients	84	243	32	359
Number of patients who experienced a first adverse event	13	40	4	57
Number of patients censored	71	203	28	302
Log rank test				0.867
Univariate cox proportional hazard model				
Hazard ratio: Carbon fiber implant vs Titanium implant				0.90 (95% CI: 0.48;1.69)
*P* value				0.743
Hazard ratio: Combination of carbon fiber and titanium implant vs titanium implant				0.78 (95% CI: 0.28;2.19)
*P* value				0.643
Multivariable cox proportional hazard model^a^				
Hazard ratio: Carbon fiber implant vs Titanium implant				0.53 (95% CI: 0.26;1.07)
*P* value				0.075
Hazard ratio: Combination of carbon fiber and titanium implant vs titnium implant				0.57 (95% CI: 0.20;1.64)
*P* value				0.300

Unadjusted hazard ratio including 95% confidence interval for treatment difference was calculated using Wald test.

^a^Adjusted hazard ratio, adjusted for sex, age, adjuvant therapy as radiation therapy treatment administration and according to the tumor group including 95% confidence interval for implant difference was calculated using Wald test.

The estimated risk of having at least one postoperative AE, including construct failures with and without loss of correction, wound infection (deep or superficial), non-union and wound dehiscence, was statistically comparable between patients receiving carbon fiber, titanium or a combination of both carbon fiber and titanium implants (*P* > 0.05). In the overall cohort, the estimated risk was 16.2 % (95% CI: 12.5-20.4). When stratified by implant group, the risk was 15.5% (95% CI: 8.5-25.0) in the carbon fiber group, 16.5% (95% CI: 12.0-21.7) in the titanium group, and 15.6% (95% CI: 5.3-32.8) in the combination group. A detailed breakdown of these estimates is provided in Table 4. Among patients who experienced at least one AE, median time to the first AE was 0.1 year, with an interquartile range of 0.0 to 0.8 years.

**Table 4.** Adverse Event Risk Analysis by Implant Group

Adverse events	Implant								*P* value
	Carbon fiber implant N = 84		Titanium implant N = 243		Combination of carbon fiber and titanium implants N = 32		Total N = 359		
	n^a^	%^b^(95% CI^c^)	n^a^	%^b^(95% CI^c^)	n^a^	%^b^(95% CI^c^)	n^a^	%^b^(95% CI^c^)	
Any adverse event	13	15.5 (8.5; 25.0)	40	16.5 (12.0; 21.7)	5	15.6 (5.3; 32.8)	58	16.2 (12.5; 20.4)	0.974^d^
Construct failure with loss of correction	5	6.0 (2.0; 13.3)	12	4.9 (2.6; 8.5)	3	9.4 (2.0; 25.0)	20	5.6 (3.4; 8.5)	0.530^e^
Construct failure without loss of correction	4	4.8 (1.3; 11.7)	7	2.9 (1.2; 5.8)	0	0.0 (0.0; 10.9)	11	3.1 (1.5; 5.4)	0.538^e^
Deep wound infection	4	4.8 (1.3; 11.7)	18	7.4 (4.4; 11.5)	1	3.1 (0.1; 16.2)	23	6.4 (4.1; 9.5)	0.507^d^
Non-union	0	0.0 (0.0; 4.3)	2	0.8 (0.1; 2.9)	0	0.0 (0.0; 10.9)	2	0.6 (0.1; 2.0)	1.000^e^
Superficial wound infection	3	3.6 (0.7; 10.1)	4	1.6 (0.5; 4.2)	0	0.0 (0.0; 10.9)	7	1.9 (0.8; 4.0)	0.442^e^
Wound dehiscence	2	2.4 (0.3; 8.3)	9	3.7 (1.7; 6.9)	1	3.1 (0.1; 16.2)	12	3.3 (1.7; 5.8)	0.897^e^

^a^Number of patients with at least one AE.

^b^Estimated risk of developing at least one AE.

^c^Confidence intervals for percentages were calculated using the Clopper Pearson method.

^d^Chi-Square test.

^e^Fisher’s exact test.

### Overall survival, Recurrence-Free Survival and Progression-Free Survival

**Figure 2.**
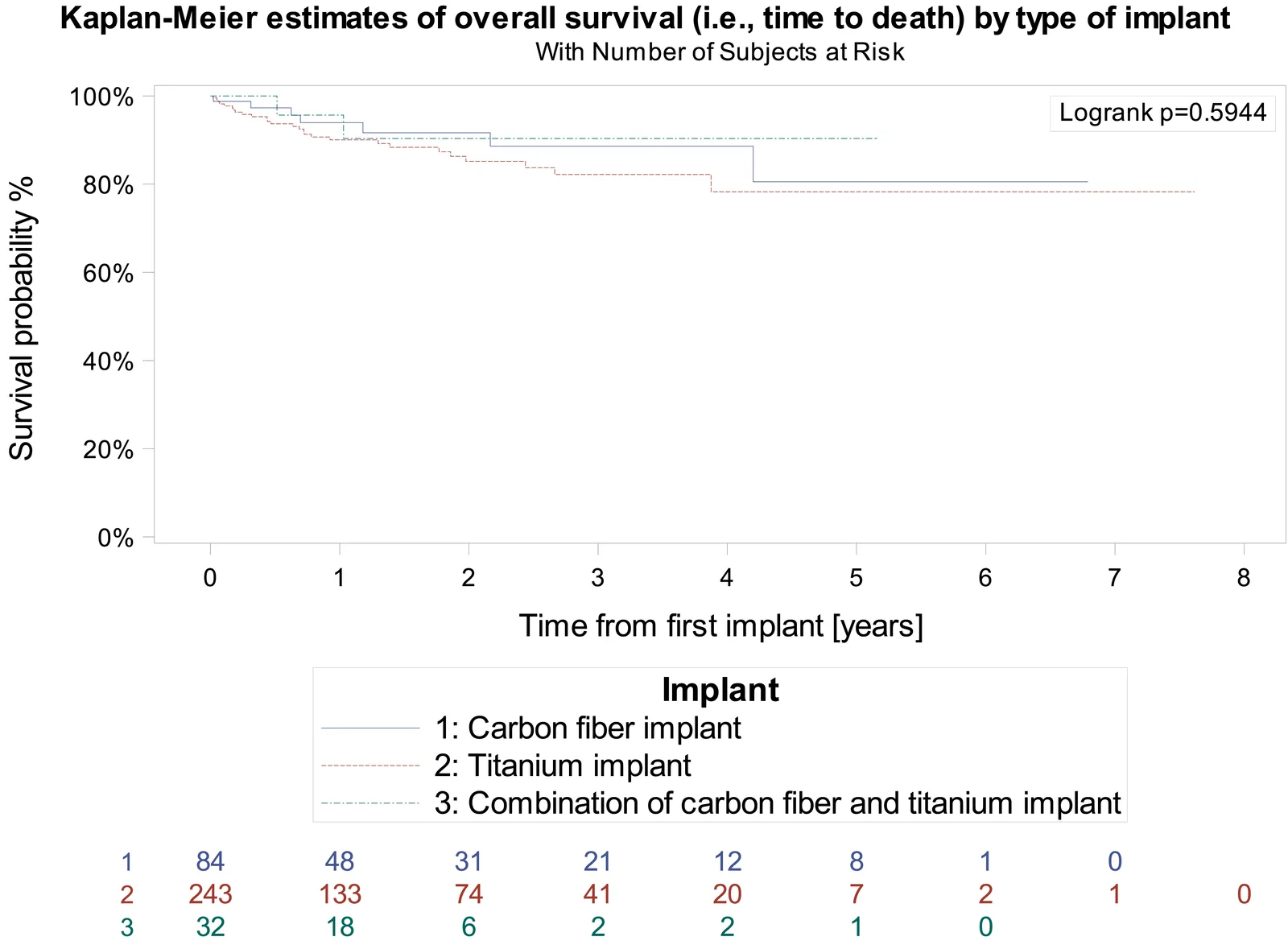
Kaplan–Meier survival curve estimating the probability of overall survival by implant type

**Figure 3.**
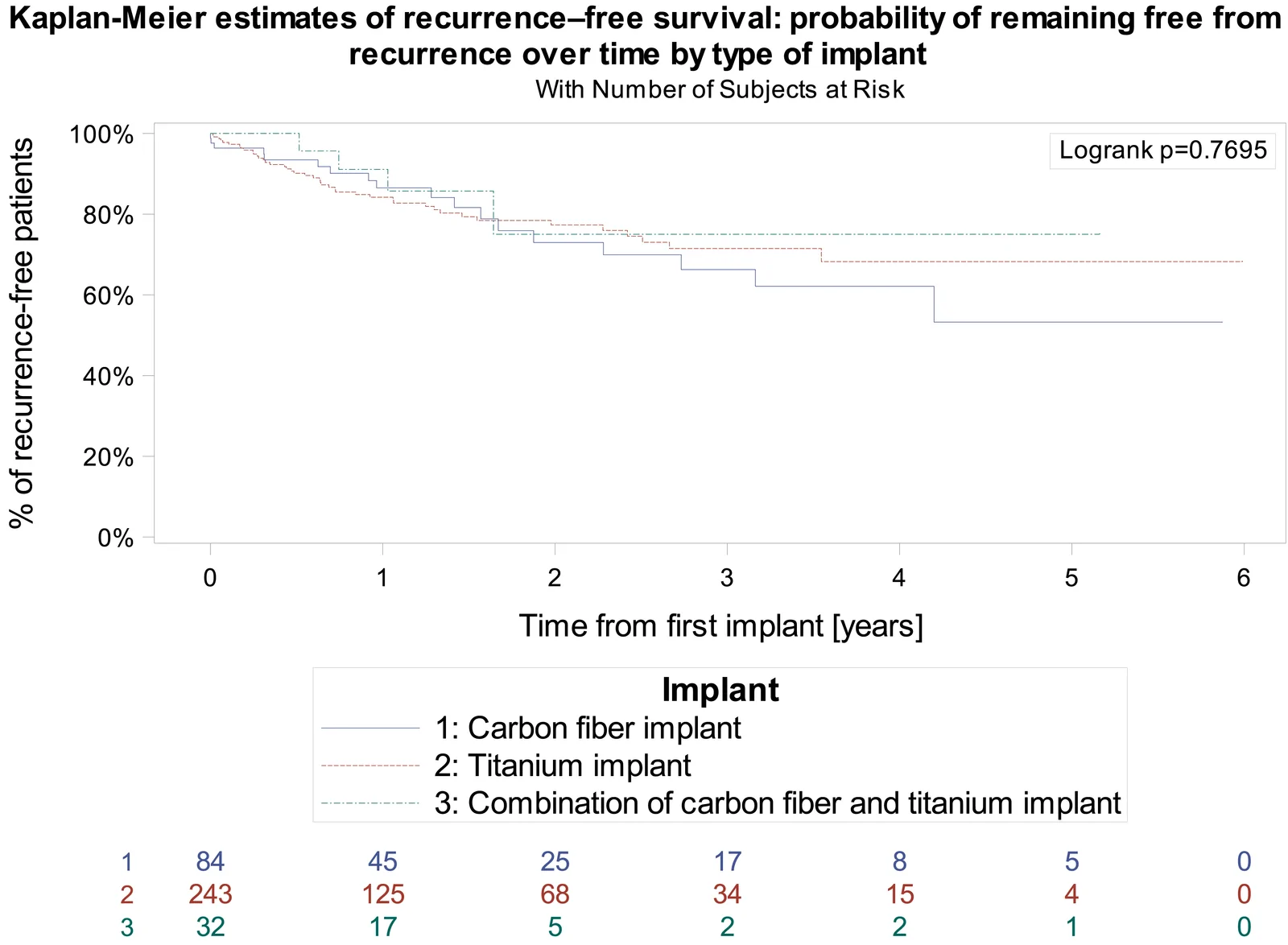
Kaplan–Meier survival curve estimating the probability of remaining free from recurrence over time by implant type using a combined endpoint of local recurrence and death

**Figure 4.**
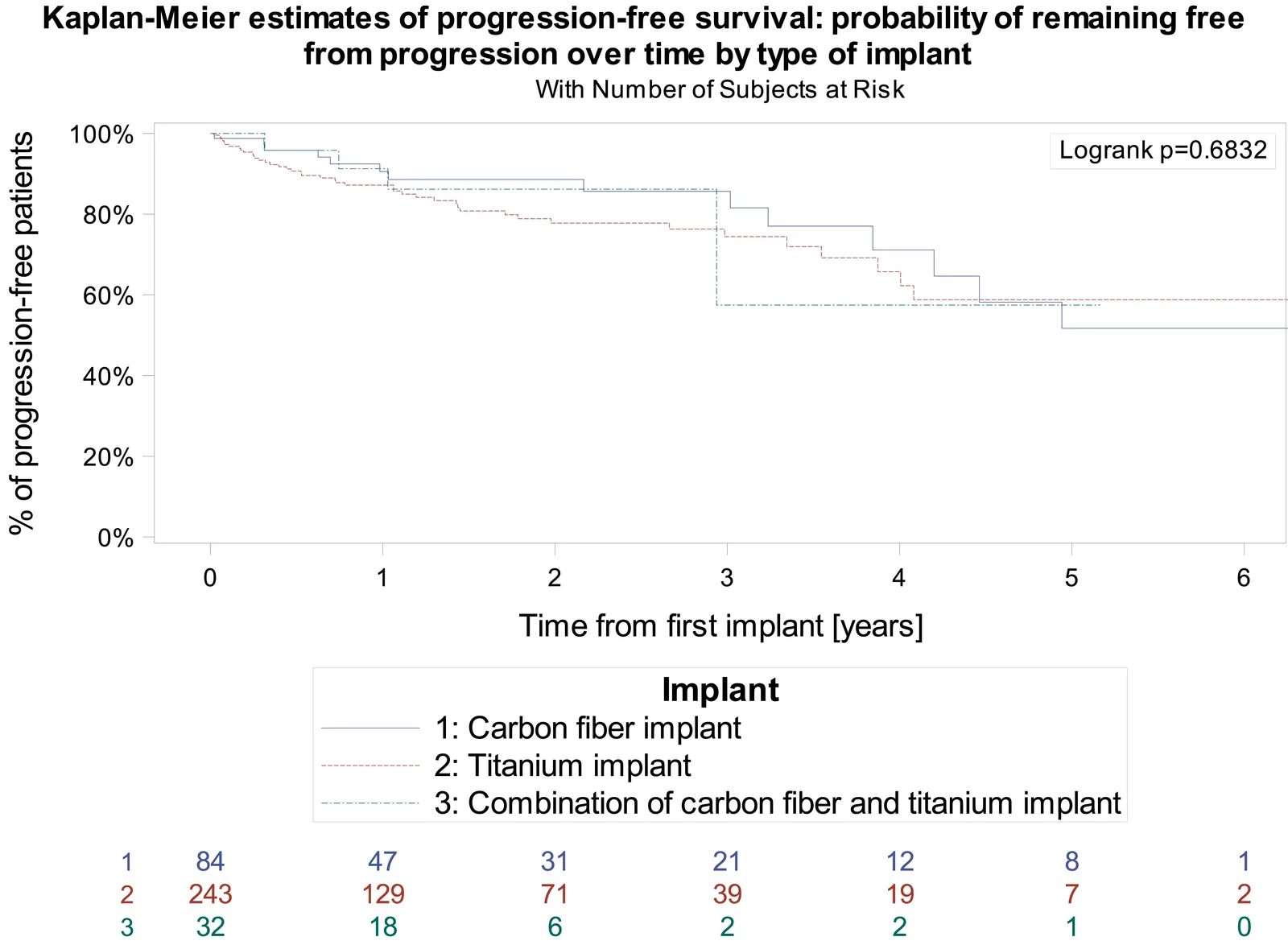
Kaplan–Meier survival curve estimating the probability of remaining progression-free over time by implant type using a combined endpoint of progression (metastases) and death

Kaplan-Meier curve analyses demonstrated no significant difference in overall survival (log-rank *P* = 0.594), recurrence-free survival with a combined endpoint (log-rank *P* = 0.770) and progression-free survival with a combined endpoint (log-rank *P* = 0.683) (Figures 2–4).

Univariate and multivariable Cox proportional hazards analyses confirmed no differences in the risk of death, local recurrence, or progression (metastases) when using combined endpoints, between patients with a carbon fiber or a combination of a carbon fiber implant compared to those with a titanium implant only.

Compared to titanium (reference), the HR for death from the multivariable Cox proportional hazards regression models were 0.69 (95% CI: 0.29;1.62, *P* = 0.39) for carbon fiber and 0.50 (95% CI: 0.12;2.16, *P* = 0.36) for combination implants (Table 5). Similarly, the HR for local recurrence were 0.93 (95% CI: 0.50;1.72, *P* = 0.82) for carbon fiber and 0.31 (95% CI: 0.07;1.30, *P* = 0.11) for combination implants. For progression (metastases), the HR were 0.80 (95% CI: 0.42;1.51, *P* = 0.48) for carbon fiber and 0.70 (95% CI: 0.26;1.92, *P* = 0.49) for combination implants (Table 6).

**Table 5.** Summary of Overall Survival by Implant Group

Outcome	Implant			
	Carbon fiber implant	Titanium implant	Combination of carbon fiber and titanium implants	Summary/*P* value
Total number of patients	84	243	32	359
Number of patients who died	7	27	2	36
Number of patients censored	77	216	30	323
Log rank test				0.594
Univariate cox proportional hazard model				
Hazard ratio: Carbon fiber implant vs Titanium implant				0.69 (95% CI: 0.30;1.58)
*P* value				0.380
Hazard ratio: Combination of carbon fiber and titanium implant vs titanium implant				0.64 (95% CI: 0.15;2.70)
*P* value				0.545
Multivariable cox proportional hazard model^a^				
Hazard ratio: Carbon fiber implant vs Titanium implant				0.69 (95% CI: 0.29;1.62)
*P* value				0.389
Hazard ratio: Combination of carbon fiber and titanium implant vs titanium implant				0.50 (95% CI: 0.12;2.16)
*P* value				0.356

Time at risk starts at the timepoint of the first implant in case of patients with a combination of carbon fiber and titanium implant or in case of patients with more than one carbon fiber or titanium implant. Time at risk ends at death, dropout or at the last follow-up visit whichever occurs first.

Patients are censored at time dropout or at their last follow-up visit whichever occurred first.

Unadjusted hazard ratio including 95% confidence interval for treatment difference was calculated using Wald test.

^a^Adjusted hazard ratio, adjusted for sex, age, adjuvant therapy as radiation therapy treatment administration and according to the tumor group including 95% confidence interval for implant difference was calculated using Wald test.

**Table 6.** Summary of Progression-free Survival by Implant Type, Using a Combined Endpoint of Progression (Metastases) and Death

Outcome	Implant			
	Carbon fiber implant	Titanium implant	Combination of carbon fiber and titanium implant	Summary/*P* value
Total number of patients	84	243	32	359
Number of patients who experienced metastasis	14	43	4	61
Number of patients censored	70	200	28	298
Log rank test				0.683
Univariate cox proportional hazard model				
Hazard ratio: Carbon fiber implant vs Titanium implant				0.78 (95% CI: 0.42;1.43)
*P* value				0.419
Hazard ratio: Combination of carbon fiber and titanium implant vs titanium implant				0.80 (95% CI: 0.29;2.22)
*P* value				0.664
Multivariable cox proportional hazard model^a^				
Hazard ratio: Carbon fiber implant vs Titanium implant				0.80 (95% CI: 0.42;1.51)
*P* value				0.487
Hazard ratio: Combination of carbon fiber and titanium implant vs titanium implant				0.70 (95% CI: 0.26;1.92)
*P* value				0.490

Time at risk starts at the timepoint of the first implant in case of patients with a combination of carbon fiber and titanium implant or in case of patients with more than one carbon fiber or titanium implant. Time at risk ends at the first timepoint of metastases, death, dropout or at the last follow-up visit whichever occurs first.

Patients are censored at time of dropout or at their last follow-up visit whichever occurred first.

Unadjusted hazard ratio including 95% confidence interval for treatment difference was calculated using Wald test.

^a^Adjusted hazard ratio, adjusted for sex, age, adjuvant therapy as radiation therapy treatment administration and according to the tumor group including 95% confidence interval for implant difference was calculated using Wald test.

Sensitivity analyses applying the event-free approach without a combined endpoint confirmed no significant differences between the implant types in the risk of local recurrence and progression (metastases).

## Discussion

This multicenter study leverages data from the PTRON database to evaluate the impact of implant material—carbon fiber vs traditional metallic constructs vs a combination of both—on hardware-related AEs and tumor control outcomes in patients undergoing surgery for primary spinal tumors. Our findings suggest that there is no statistically significant difference among carbon fiber, titanium, and combined carbon fiber–titanium implants in the overall risk of experiencing at least one AE, in the probability of remaining free from AEs over time, or in the hazard of experiencing a first AE. Moreover, there was no difference in overall survival, recurrence-free survival and progression-free survival across all implant types.

In our cohort, the risk of developing at least one hardware-related AE remained low and comparable irrespective of implant choice. Specifically, AEs such as construct failure (with or without loss of correction), wound complications, and non-union occurred at similar rates in patients treated with carbon fiber vs titanium implants. The risk of developing at least one hardware failure in our cohort groups was comparable to the incidence reported by other authors in literature. Yee at al.^9^ in their retrospective study on survival and the incidence of fusion, implant related failures after surgery for spinal metastasis, reported results on decompression and fusion performed on metastatic patients using titanium instrumentation. The estimated cumulative incidence of hardware failure was 4.2%. The safety profile observed for carbon fiber implants is particularly relevant given their increasing use in spine oncology.^4,6,7,10^ This theoretical advantage has led to growing interest in the spinal oncology community, particularly for the treatment of non-resectable primary spinal tumors or resectable lesions where surgery carries a high risk of severe functional sequelae for the patient, as it enhances the potential for productive collaboration between surgeons and radiation oncologists by improving imaging for postoperative planning and radiotherapy delivery. This evolution in implant materials could improve the oncologic outcomes and potentially lead to the adoption of the separation surgery concept in the management of primary spinal tumors—an approach previously reserved almost exclusively for metastatic disease.^5,11^

Regarding tumor control, our secondary analysis demonstrated no statistically significant difference in overall survival, recurrence-free survival, or progression-free survival between implant types. Kaplan-Meier curve analyses and multivariable Cox regression models adjusting for tumor type, age, sex, and adjuvant radiation therapy showed no differences in overall survival, recurrence-free and progression-free survival nor in the risk of death, local recurrence and progression (metastases) between the implant groups. These data confirm the results reported by Ward J et al 1^12^ in their study on the impact of instrumentation material on local recurrence in the treatment of spinal metastasis with separation surgery followed by stereotactic body radiotherapy (SBRT). In this study the authors analyzed 263 patients who underwent spinal decompression and fusion, for which 148 patients met predetermined inclusion criteria. Of these, 49 had titanium instrumentation, and 99 had CFR-PEEK. Complication profiles, including hardware failure and infection were similar between the groups. There was no significant difference in progression-free survival between all CFR-PEEK and titanium patients. Interestingly when comparing patients in which recurrence was noted, CFR-PEEK patients had recurrence detected two times earlier than titanium patients (94 days vs 189 days; *P* = 0.013), which may reflect the enhanced imaging properties of carbon fiber implants which could facilitate earlier tumor recurrence. While these results pertain to metastatic disease, they could support the broader principle that radiolucent implants may enable earlier detection of progression (metastases)—an advantage that could be particularly relevant in the surveillance of primary spinal tumors as well, although the biological behavior and clinical context differ.

This study has several limitations that warrant consideration. First, the analysis is based on a non-randomized, observational cohort derived from a multicenter registry, which introduces the potential for selection bias in implant choice. Implant selection in spinal oncology is influenced by multiple factors. Surgeons are more likely to use carbon fiber constructs in cases requiring high-quality imaging surveillance (eg, chordomas) or when postoperative radiotherapy is anticipated, as carbon fiber minimizes imaging artifacts and facilitates treatment planning. Titanium implants remain common in patients with poor bone quality, long constructs, or when immediate mechanical strength is prioritized. Mixed constructs may be selected in complex reconstructions to combine the mechanical benefits of titanium with the radiological advantages of carbon fiber. In addition, the localization of the tumor within the vertebra plays a critical role. According to the Weinstein–Boriani–Biagini (WBB) staging system, tumors involving both pedicles or those with a wide extravertebral or epidural component are considered unresectable with wide margins, thereby influencing surgical planning and implant choice.^11^ Second, despite adjustments for key confounding variables, namely sex, age, adjuvant radiation therapy, and tumor type, residual confounding cannot be excluded. Notably, osteoporosis or the presence of spinal deformity data were not uniformly available and may have influenced both implant selection and risk of hardware failure. The current median follow-up of 1.9 years is insufficient to capture late implant-related adverse events, such as non-union or delayed hardware failure. This limitation is partly due to the fact that systematic documentation of implant material in the PTRON database began only recently. As a result, several complications—particularly fatigue-related construct failure or hardware failure occurring years after surgery—may not yet be observable within our cohort. A larger sample size combined with longer-term follow-up will be necessary to more reliably assess the durability of carbon fiber constructs compared with titanium. Consequently, our findings regarding long-term mechanical performance should be considered preliminary and will require confirmation in future studies with extended surveillance. A potential benefit of carbon fiber implant is the increased ability of radiation delivery which could translate in better local control rates, but our relatively short follow-up combined with our low numbers of patients who had radiation therapy and experienced a recurrence prevent us from drawing conclusion on local control. Finally, heterogeneity in surgical techniques, tumor pathology, and adjuvant treatment strategies across institutions may affect the generalizability of the findings. Nonetheless, the multicenter design and real-world nature of the data enhance the external validity and clinical relevance of this study.

## Conclusion

In this multicenter analysis of patients with primary spinal tumors, the use of carbon fiber spinal implants demonstrated a comparable safety and oncologic profile to traditional metallic instrumentation. The risk for hardware-related AEs did not significantly differ between implant types, even when adjusted for confounding variables. Similarly, overall survival, recurrence-free survival and progression-free survival outcomes were equivalent across groups.

Beyond clinical equivalence, carbon fiber implants offer significant radiological advantages that enhance postoperative imaging, facilitate radiation therapy planning, and promote multidisciplinary coordination. Finally, the economic implications of carbon fiber instrumentation should be acknowledged. Although carbon fiber implants are associated with higher initial costs compared to traditional titanium constructs, their potential benefits—such as facilitating earlier recurrence detection, enabling more precise radiotherapy delivery, and reducing the need for repeat imaging—may provide long-term value. Formal cost-effectiveness analyses will be needed to establish whether these theoretical advantages translate into measurable healthcare savings.

Taken together, these findings support the safe and effective incorporation of carbon fiber implants into the spine oncology armamentarium, but findings are preliminary, may be subject to type II error, and longer-term follow-up is required to confirm equivalence between implant types.
