# Erratum to “Utilization of Carbon Fiber Spinal Instrumentation in Primary Spinal Tumors: Outcome Analysis from the AO Spine Primary Tumor Research and Outcomes Network”

**DOI:** 10.1177/21925682261496904

**Published:** 2026-10-11

Evangelisti G, Wei F, Gasbarrini A, et al. Utilization of carbon fiber spinal instrumentation in primary spinal tumors: outcome analysis from the AO Spine Primary Tumor Research and Outcomes Network [published online ahead of print September 13, 2026]. *Global Spine J*. doi: 10.1177/21925682261417978

In this article, Marie-Laure Vial was incorrectly included as the second author in the initial OnlineFirst version. The article has been updated to reflect the correct author list. The corrected author byline is provided below:

Gisberto Evangelisti, Feng Wei, Alessandro Gasbarrini, Laurence D. Rhines, Ziya L. Gokaslan, Jeremy Reynolds, Alessandro Luzzati, Alexander C. Disch, Praveen V. Mummaneni, Daniel G. Tobert, Michelle J. Clarke, Aron Lazary, Chetan Bettegowda, Riccardo Cecchinato, Stefano Boriani, Ori Barzilai, Cordula Netzer, Arjun Sahgal, Jorrit-Jan Verlaan, Charles G. Fisher, Ilya Laufer, Nicolas Dea, on behalf of AO Spine Knowledge Forum Tumor.

